# *Trypanosoma equiperdum* Low Molecular Weight Proteins As Candidates for Specific Serological Diagnosis of Dourine

**DOI:** 10.3389/fvets.2018.00040

**Published:** 2018-03-05

**Authors:** Mirella Luciani, Tiziana Di Febo, Massimiliano Orsini, Ivanka Krasteva, Angela Cattaneo, Michele Podaliri Vulpiani, Chiara Di Pancrazio, Angela Bachi, Manuela Tittarelli

**Affiliations:** ^1^Istituto Zooprofilattico Sperimentale dell’Abruzzo e del Molise G. Caporale, Teramo, Italy; ^2^Istituto FIRC di Oncologia Molecolare (IFOM), Milan, Italy

**Keywords:** *Trypanosoma equiperdum*, dourine, immunoblotting, mass spectrometry, protein identification

## Abstract

The diagnosis of dourine can be difficult because the clinical signs of this disease in horses are similar to those of surra, caused by *Trypanosoma evansi*. Moreover, *T. equiperdum* and *T. evansi* are closely related and, so far, they cannot be distinguished using serological tests. In a previous work, the *T. equiperdum* protein pattern recognized by antibodies from dourine-infected horses and the humoral immune response kinetics were investigated by immunoblotting assay; a total of 20 sera from naturally and experimentally infected horses and from healthy animals were tested. Immunoblotting analysis showed that antibodies from infected horses specifically bind *T. equiperdum* low molecular weight proteins (from 16 to 35 kDa), which are not recognized by antibodies from uninfected horses. In this work, we tested other 615 sera (7 from naturally infected horses and 608 sera from healthy horses and donkeys): results confirmed the data obtained previously. In addition, six SDS-PAGE bands with molecular weight ranging from 10 to 37 kDa were analyzed by mass spectrometry, in order to identify immunogenic proteins that could be used as biomarkers for the diagnosis of dourine. A total of 167 proteins were identified. Among them, 37 were found unique for *T. equiperdum*. Twenty-four of them could represent possible candidate diagnostic antigens for the development of serological tests specific for *T. equiperdum*.

## Introduction

*Trypanosoma equiperdum* is the causative agent of dourine, a chronic or acute contagious disease of equids. Dourine is the only sexually transmitted trypanosomosis and does not involve invertebrate vectors. *T. equiperdum* is different from other trypanosomes since it is mainly detected in the host tissues and only occasionally in the blood. There are no known natural reservoirs of the parasite other than infected equids (1).

*Trypanosoma equiperdum* is closely related to other trypanosomes of the subgenus *Trypanozoon*, as *T. evansi*, the causative agent of surra, and *T. brucei*, the agent of nagana (2, 3). They have proteins with a high degree of similarity, such as cytoskeletal proteins, that are responsible of a strong and cross-reactive humoral immune response (1). The observation of clinical signs and lesions, the isolation and the identification of the parasite, and serological investigations are used in parallel in the diagnosis of dourine. However, in some cases, it is difficult to identify clinical signs and lesions or there could be confusions with other diseases, such as surra, that give rise to similar clinical signs. Moreover, *T. equiperdum* morphology and motility are very similar to those of other species of the subgenus *Trypanozoon*, and in particular to *T. evansi* (1). Recently, according to phylogenetic analysis, some authors have suggested that *T. equiperdum* and *T. evansi* may have evolved from *T. brucei* and should be considered as their subspecies (*T. brucei equiperdum* and *T. b. evansi*) (4, 5). Serological tests, as complement fixation test (CFT), indirect fluorescent antibody test (IFAT), and ELISA, cannot distinguish between dourine and surra (1, 2). To date, serodiagnosis of dourine is carried out using the whole *Trypanosoma* antigen and polyclonal secondary antibodies, so there are currently no available serological tests specific for dourine (1). Dourine serodiagnosis will be improved only using selected *T. equiperdum* recombinant proteins and monoclonal antibodies.

In the same way, no *T. equiperdum*-specific polymerase chain reaction (PCR) method is currently available. Recently, some authors used a *Trypanozoon*-specific real-time PCR for the detection of *T. equiperdum* DNA in tissues and fluid samples and low numbers of trypanosomes were detected (6, 7). However, the identification of trypanosomes in blood samples by PCR and other similar DNA amplification methods could be difficult, in particular after the initial phase of the infection (1, 8).

In our previous work (9), a chemiluminescent immunoblotting assay (cIB) was developed and used to study *T. equiperdum* antigen patterns recognized by serum antibodies from uninfected and infected animals. A total of 20 sera (8 from naturally infected horses, 2 from experimentally infected mares, and 10 from healthy control animals) were tested. Moreover, we tested seven serum samples previously obtained from an animal experimentally infected by transfusion of blood collected from another dourine-positive horse. Results revealed that antibodies from infected horses specifically bind to *T. equiperdum* low molecular weight bands ranging from 16 to 35 kDa, in contrast to antibodies from healthy horses that recognize only bands with molecular weight >37 kDa.

In the present work, we applied the cIB for testing a greater number (615) of sera, to confirm results previously obtained. We also analyzed by mass spectrometry six SDS-PAGE bands with molecular weight ranging between 37 and 10 kDa, in order to identify proteins only recognized by antibodies from infected horses. The identification of *T. equiperdum* proteins involved in horse immune response during the infection is important to find potential biomarkers and produce recombinant proteins that could be used, as specific antigens, in the differential diagnosis of dourine.

## Materials and Methods

### Sera

Sera from 608 healthy animals (549 horses and 59 donkeys) were collected in Northern Italy regions. Seven sera from naturally infected horses were collected in the field: one from an outbreak in Basilicata region (Southern Italy), five obtained from Namibia, and one from a German farm (pony imported from Mongolia).

All sera were tested for dourine by CFT and IFA according to the OIE Manual of Diagnostic Tests and Vaccines (1) using the Onderstepoort Veterinary Institute strain of *T. equiperdum* (OVI *T.e*.) as antigen, produced according to the OIE manual (1). Origin and antibody titer of tested sera are shown in Table 1.

**Table 1:** Immunoblotting test for dourine.

Origin (country/region/province)	Number of sera	CFT titer^a^	IFA titer^b^
Italy (Bolzano)	41	<1:5	<1:80
Italy (Emilia Romagna)	95	<1:5	<1:80
Italy (Friuli Venezia Giulia)	30	<1:5	<1:80
Italy (Lombardia)	7	<1:5	<1:80
Italy (Piemonte)	25	<1:5	<1:80
Italy (Sardegna)	150	<1:5	<1:80
Italy (Toscana)	4	<1:5	<1:80
Italy (Trento)	107	<1:5	<1:80
Italy (Veneto)	149	<1:5	<1:80
Italy (Basilicata)	1	1:160	1:320
Germany	1	1:40	1:320
Namibia	5	1:20	1:80
		1:10	1:80
		1:160	1:640
		1:10	1:80
		1:320	1:640

Total	615		

*Origin and CFT/IFA titer of the sera tested*.

*^a^CFT negative <1:5*.

*^b^IFA negative <1:80*.

### Immunoblotting

Onderstepoort Veterinary Institute strain of *T. equiperdum* antigen was purified from rat blood as described in Ref. (9). Animal experimentation was done according to Italian national law (Legislative Decree 26/2014) (10) and Directive 2010/63/EU on the protection of animals used for scientific purposes (11). Ethical approval was obtained from the Italian Ministry of Health (Protocol no. 114/2014 PR of 19.12.2014, ex Lgs. D. 26/2014, art. 31).

Immunoblotting (cIB) was performed according to Ref. (9), using the purified OVI *T.e*. antigen and NuPage^®^ 12% Bis-Tris pre-cast gels (Life Technologies, Paisley, UK) at 200 V. OVI *T.e*. proteins were then transferred onto a nitrocellulose membrane. After blocking with skim milk, membranes were cut into strips, which were incubated with sera diluted 1:10 and then with a monoclonal antibody anti-horse IgG-HRP-conjugate (MAb IZSA&M, Italy).

Antigen-antibody reactions were visualized by adding the Amersham^TM^ ECL Select^TM^ Western Blotting Detection Reagent (GE Healthcare, Uppsala, Sweden). Images were acquired using the ChemiDoc MP (Bio-Rad) and the Image Lab Software, version 4.0 (Bio-Rad); detection time ranged from 1 to 5 s, for both positive and negative sera. As molecular weight marker, BenchMark^TM^ Prestained Protein Ladder (Life Technologies) was used.

### Mass Spectrometry Analysis (nLC-ESI-MS/MS)

Onderstepoort Veterinary Institute strain of *T. equiperdum* proteins were quantified using Coomassie Plus (Bradford) Assay Kit (Thermo Scientific, Rockford, IL, USA) and separated using a NuPage^®^ 12% Bis-Tris pre-cast gel (Life Technologies) (4.5-µg proteins per well) at 200 V. Then proteins were stained overnight with SimplyBlue SafeStain (Life Technologies). Stained gel was stored in deionized H_2_O at +4°C until protein analysis.

**Figure 1:**
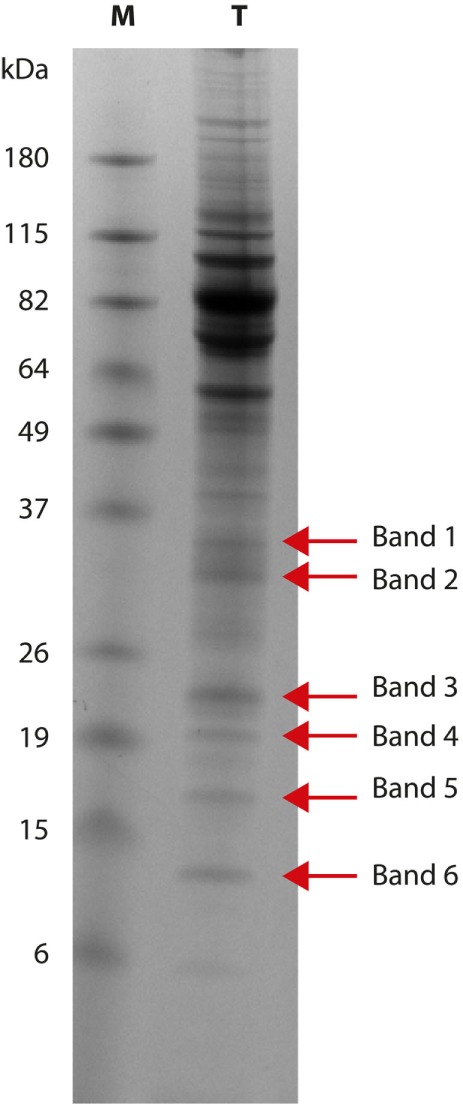
*Trypanosoma equiperdum* OVI SDS-PAGE. Protein pattern (lane T) obtained using a 12% polyacrylamide gel: red arrows show the six bands analyzed by mass spectrometry. Lane M: molecular weight marker (BenchMark Prestained Protein Ladder, Life Technologies).

Six bands with molecular weight ranging from 37 to 10 kDa (Figure 1) were cut from the gel; proteins were then destained using the standard in-gel protocol. Reduction with 10-mM DTT, alkylation with 55-mM IAA, and trypsin digestion were carried out as previously reported (12, 13). After acidification with 0.1% formic acid, peptide mixtures were concentrated and desalted on homemade StageTips C18 (14, 15). Peptides were injected on an UPLC EASY-nLC 1000 (Thermo Scientific) and separated on a homemade fused silica capillary column (75-µm i.d., length 25 cm), packed in house with ReproSil-Pur C18-AQ 1.9-µm beads (Dr. Maisch, Ammerbuch-Entringen, Germany). A gradient of eluents A (2% acetonitrile, 0.1% formic acid) and B (80% acetonitrile with 0.1% formic acid) was used to achieve separation, from 5 to 100% B (in 30 min, 250 nL/min flow rate). The nLC system was connected to a quadrupole Orbitrap QExactive-HF mass spectrometer (Thermo Fisher) equipped with a nanoelectrospray ion source (Proxeon Biosystems). MS data were acquired using a data-dependent top 15 method for HCD fragmentation. Survey full scan MS spectra (300–1,750 Th) were acquired in the Orbitrap with 60,000 resolution, AGC target 1^e6^, and IT 120 ms. For HCD spectra, resolution was set to 15,000 at *m*/*z* 200, AGC target 1^e5^, and IT 120 ms: NCE 28% and isolation width 3.0 *m*/*z*. Raw data were processed with Proteome Discoverer (version 1.4.1.14, Thermo Scientific) and Mascot (version 2.6.0, Matrix Science) searching against the 7,668 *T. equiperdum* OVI protein sequences annotated in DDBJ/EMBL/GenBank under the accession number CZPT02000000 (16), assuming a fragment ion mass tolerance of 20 ppm and a parent ion tolerance of 10 ppm; specified enzyme was trypsin; carbamidomethylation of cysteine was set as a fixed modification; oxidation of methionine and acetylation of the N-terminus of proteins were set as variable modifications. Scaffold (version 4.4.3, Proteome Software Inc., Portland, OR, USA) was used to validate MS/MS-based peptide and protein identifications. Peptide identifications were accepted when probability, calculated using Scaffold Local FDR algorithm, was greater than 95%. Protein identifications were accepted when probability was greater than 99% and proteins contained at least 3 identified peptides. Protein probabilities were assigned by the Protein Prophet algorithm (17). Proteins that share similar peptides and could not be differentiated based on MS/MS analysis alone were grouped to satisfy the principles of parsimony.

### Bioinformatic Analysis

Functional features for proteins identified by mass spectrometry analysis were derived from the correspondent Uniprot entries (18). They were further annotated in terms of topological and immunological features. Namely, presence and localization of transmembrane helices and signal peptides were predicted by THMM (19) and signalP (20), respectively. Subcellular localization was predicted by using the TargetP software (21), while B-cell linear epitopes were predicted by Bepi-pred (22) imposing a threshold equal or >0.6 and a minimum length of four residues. Results from each predictor were parsed and ranked by *ad hoc* developed python scripts, when necessary. Finally, protein conservation was assessed by Blasting (23) them against all Uniprot entries belonging to the *Trypanosoma* genus.

## Results

### Immunoblotting

Serum antibodies from non-infected animals identified common bands ranging from 37 to 180 kDa. The antigen pattern of OVI *T.e*. recognized by antibodies from infected horses showed the presence of low molecular weight bands ranging from 16 to 35 kDa. In particular, positive sera reacted with the following bands: 35 kDa (5 sera out of 7), 30 kDa (5 sera out of 7), 28 kDa (3 sera out of 7), 26 kDa (4 sera out of 7), 21/22 kDa (3 sera out of 7), 19 kDa (7 sera out of 7), and 16 kDa (3 sera out of 7). None of the bands with molecular weight lower than 16 kDa were recognized by positive sera. These results confirmed data published in Ref. (9).

### Mass Spectrometry (nLC-ESI-MS/MS) and Bioinformatics Analysis

Six *T. equiperdum* protein bands with molecular weights of 35 kDa (band 1), 30 kDa (band 2), 21/22 kDa (band 3), 19 kDa (band 4), 16 kDa (band 5), and 10 kDa (band 6) were selected for mass spectrometry analysis (Figure 1). Bands from 1 to 5 were recognized by antibodies of infected horses; band 6 was not recognized by the positive sera tested.

According to selected validation criteria (peptide thresholds: 95.0%; protein thresholds: 99.0%; minimum three peptides/protein), 167 trypanosoma-specific proteins were identified in five out of the six bands analyzed. Forty-six proteins were found in more than one band (Table S1 in Supplementary Material). In band 5, no trypanosoma-specific proteins were identified.

Annotation was successfully for all proteins in the dataset. The most represented categories were ribosomal proteins (*n* = 38) and integral components of membrane (*n* = 23), followed by protein-binding nucleosides (ATP or GTP, *n* = 19), proteins involved in motility or in DNA binding. Several additional categories were identified although with lower occurrence, while no category was assigned for 37 proteins. Twenty-one proteins were tagged as “uncharacterized”; however, for four of them it was possible to assign gene ontology and topology features. Predictions for the presence of transmembrane domains, peptide signals, and B-cell epitopes are shown in Table S2 in Supplementary Material.

Identified proteins were compared, by similarity searching, to all known proteins within the *Trypanosoma* genus, in order to establish their conservation. A total of 37 out of 167 proteins resulted in having unique sequence; the remaining 130 proteins showed full identity with proteins of *T. evansi, T. brucei brucei, T. b. gambiense, T. b. rhodesiense*, and *T. congolense* (Table S2 in Supplementary Material). Relaxing comparison threshold to 90% of similarity and 90% of reciprocal horizontal coverage, eight of them (SCU66692.1, SCU67526.1, SCU67064.1, SCU66719.1, SCU71330.1, SCU69415.1, SCU68469.1, SCU70408.1) still gave no matches with other *Trypanosoma* proteins (Table S2 in Supplementary Material). Thirty-six out of the 167 identified proteins had a molecular weight >37 kDa and they were excluded from the list of possible diagnostic candidate antigens. In fact, the presence of these protein fragments <37 kDa could be due to proteolytic cleavage of higher molecular weight native proteins, occurred during OVI *T.e*. antigen purification and preparation for SDS-PAGE, carried out without using protease inhibitors. This was confirmed by Expasy PeptideCutter analysis that predicted several potential cleavage sites for proteases or chemicals in the sequence of all the proteins. The 24 unique proteins with molecular weight lower than 37 kDa could represent candidate antigens for diagnostic assays. Nine of them (SCU66719.1, SCU68469.1, SCU67625.1, SCU66039.1, SCU66661.1, SCU67727.1, SCU67836.1, SCU69999.1, SCU65896.1) showed the presence of potential B-cell epitopes in a fraction higher or equal to 30%. Specifically, for the peptidyl-prolyl cis-trans isomerase (SCU68469.1) and the ALBA-Domain protein (SCU66039.1) the percentage of B-cell epitopes resulted of 45.8 and 60.4%, respectively. The recombinant *T. cruzi* 24-kDa flagellar calcium-binding protein has been found immunogenic and was used as antigen in the diagnosis of Chagas’ disease with high degrees of diagnostic sensitivity and specificity (24–26). Other proteins such as peptidyl-prolyl cis-trans isomerase, ubiquitin-conjugating enzyme E2, 60S ribosomal proteins, and 40S ribosomal proteins were detected in plasma from African sleeping sickness patients infected with *T. b. rhodesiense* (27) and/or in the secretome of *T. congolense* (28) and their diagnostic value should be further explored.

## Discussion

Dourine was first eradicated in Italy in the 1940s, but some outbreaks were observed between the 1970s and 1980s (29) and few cases were reported at the end of the 1990s. In 2011, new outbreaks involving Sicily, Campania, and Puglia regions occurred (6, 8). All the outbreaks were caused by *T. equiperdum* and were related to sexual transmission (6, 8, 30).

In our previous work (9), 10 sera from *T. equiperdum* infected horses and 10 sera from healthy horses were tested by immunoblotting. Results showed that antibodies from infected and healthy animals reacted with bands with molecular weight ranging from 180 to 37 kDa; moreover, positive sera reacted also with low molecular weight bands, ranging from 35 to 16 kDa, that were not recognized by negative sera. In the present work, we tested a higher number of negative sera (608) in order to validate the immunoblotting test and to confirm results previously obtained. We also tested other seven positive sera from naturally infected horses. The protein bands 35, 30, 28, 26, 21/22, 19, and 16 kDa were recognized by three or more of the tested sera. These results confirmed those described in Ref. (9): in fact the sera tested previously recognized the same bands, except from 28 and 21/22 kDa bands. We could not analyze a greater number of positive sera, due to the difficulties to collect sera from non-Italian dourine outbreaks. After 2012, in Italy there were no other confirmed clinical cases of dourine. Moreover, in order to know which *T. equiperdum* proteins are involved in the immune response in equids, it is necessary to test sera from animals certainly positive only for dourine. In some areas, as Africa or South America, more than one *Trypanosoma* species is present, so it is difficult to distinguish between infections caused by *T. equiperdum* from those produced by other species (*T. evansi, T. congolense, T. b. brucei*) and also coinfections could be possible.

The availability of the genome sequences of *T. brucei* (31) and *T. cruzi* (32) has opened the way for transcriptome and proteome analyses of Trypanosomatids and for the production of recombinant proteins to improve serological diagnosis of human illnesses caused by these two parasites (33, 34). In 2017, the genome sequence of *T. equiperdum* was also published (16). Consequently, the identification of *T. equiperdum* proteins and the study of their characteristics and functions have become possible.

The aim of this study was the identification of immunogenic proteins recognized by antibodies of infected equid sera, in order to find potential biomarkers useful as antigens for new recombinant diagnostic tests specific for *T. equiperdum*. Six bands with molecular weight of 35, 30, 21/22, 19, 16, and 10 kDa were selected and analyzed by mass spectrometry. Bands from 1 to 5 were recognized by antibodies from positive sera. The strong intensity observed at SDS-PAGE for band 6 (10 kDa) induced us to further investigations, despite it was not recognized by our panel of sera from dourine-infected horses.

A total of 167 proteins were identified and their functional and structural features were derived by bioinformatic analysis. Thirty-seven proteins out of 167 were found unique for *T. equiperdum*, after a comparison with all sequenced proteins of other *Trypanosoma* species (*T. evansi, T. brucei brucei, T. b. gambiense, T. b. rhodesiense, T. congolense, T. cruzi, T. rangeli*, and *T. vivax*). Twenty-four of them could represent candidate antigens to design diagnostic tests specific for *T. equiperdum*, due to the uniqueness of their primary sequence within the genus *Trypanosoma*. These proteins should be produced as recombinant proteins and tested with a panel of horse sera to verify its diagnostic performances, in order to distinguish between horses infected with *T. equiperdum* and those infected with *T. evansi*.

Thirty-six identified proteins had molecular weight >37 kDa; their presence in the analyzed bands could be explained by proteolytic degradation of their native protein structure. They were not included in the list of possible diagnostic candidates, although 13 of them resulted unique for *T. equiperdum*, because their native form could occur in the high molecular weight bands recognized by both positive and negative sera. Anyway, the cross-reactions of these 13 proteins with negative sera should be investigated; in fact some of them, such as the heat shock 70 kDa proteins, have been identified as potential diagnostic reagents by other authors (26, 33, 34).

In the present work, only six bands with molecular weight ranging from 37 to 10 kDa were analyzed by mass spectrometry. Further studies should be carried out to identify proteins in the remaining bands with molecular weight <37 kDa, in order to find other *T. equiperdum* unique proteins that could be used as specific reagents in the differential diagnosis of dourine. Our western blotting was carried out in denaturing conditions and the immunogenicity of the single native proteins identified needs to be assessed in future studies in order to select the best diagnostic antigens.

## Ethics Statement

Animal experimentation was done according to Italian national law (Legislative Decree 26/2014) and Directive 2010/63/EU on the protection of animals used for scientific purposes. Ethical approval was obtained from the Italian Ministry of Health (Protocol no. 114/2014 PR of 19.12.2014, ex Lgs. D. 26/2014, art. 31).

## Author Contributions

ML and TF have made substantial contributions to the conception and design of the work; acquisition, analysis, and interpretation of data for the work; drafting the work and revising it critically for important intellectual content; final approval of the version to be published; and agreement to be accountable for all aspects of the work in ensuring that questions related to the accuracy or integrity of any part of the work are appropriately investigated and resolved. AB, AC, CP, IK, MO, MV, and MT contributed to the acquisition and analysis of data for the work; revising the work critically for important intellectual content; final approval of the version to be published; and agreement to be accountable for all aspects of the work in ensuring that questions related to the accuracy or integrity of any part of the work are appropriately investigated and resolved.

## Conflict of Interest Statement

The authors declare that the research was conducted in the absence of any commercial or financial relationships that could be construed as a potential conflict of interest.

## Supplementary Material

Table S1 *Trypanosoma equiperdum* proteins identified by mass spectrometry. Proteins listed in italic type were found in more than one band.

Table S2 Identified *Trypanosoma equiperdum* proteins listed according to their amino-acid sequence conservation (100% coverage/100% similarity) with all known proteins within the *Trypanosoma* genus. Proteins listed in bold type resulted unique for *T. equiperdum*; among unique proteins, those underlined have molecular weight <37 kDa.
